# Qualitative multi-stakeholder evaluation of the adoption, implementation and sustainment of the school-based dietary intervention “Jump-in”

**DOI:** 10.1186/s12889-024-18814-1

**Published:** 2024-05-17

**Authors:** Froukje E. Takens, Indira Indyk, Mai J. M. Chinapaw, Joanne K. Ujčič-Voortman, Femke van Nassau, Vincent Busch

**Affiliations:** 1 Department of Public and Occupational Health, Amsterdam UMC, Vrije Universiteit Amsterdam, De Boelelaan 1117, Amsterdam, The Netherlands; 2 Amsterdam Public Health Research Institute, Health Behaviour and Chronic Diseases, Amsterdam, The Netherlands; 3 Department Gezond Leven, Public Health Service of Amsterdam, Team Sarphati Amsterdam, Nieuwe Achtergracht 100, 1018 WT Amsterdam, The Netherlands

**Keywords:** School-based, Nutrition policy, Dietary behavior, Implementation evaluation, Qualitative, Children

## Abstract

**Background:**

Comprehensive school-based programs applying the WHO Health Promoting School Model have the potential to initiate and sustain behavior change and impact health. However, since they often include intervention efforts on a school’s policies, physical environment, curriculum, health care and involving parents and communities, they significantly ‘intrude’ on a complex system that is aimed primarily at education, not health promotion. More insights into and concrete strategies are therefore needed regarding their adoption, implementation, and sustainment processes to address the challenge to sustainable implementation of HPS initiatives in a primarily educational setting. This study consequently evaluates adoption, implementation and sustainment processes of Amsterdam’s Jump-in healthy nutrition HPS intervention from a multi-stakeholder perspective.

**Methods:**

We conducted semi-structured interviews and focus groups with all involved stakeholders (*n* = 131), i.e., Jump-in health promotion professionals (*n* = 5), school principals (*n* = 7), at-school Jump-in coordinators (*n* = 7), teachers (*n* = 20), parents (*n* = 50, 9 groups) and children (*n* = 42, 7 groups) from 10 primary schools that enrolled in Jump-in in the school year 2016–2017. Included schools had a higher prevalence of overweight and/or obesity than the Dutch average and they were all located in Amsterdam’s low-SEP neighborhoods. Data were analyzed using a directed content analysis, in which the Determinants of Innovation Model was used for obtaining theory-based predetermined codes, supplemented with new codes emerging from the data.

**Results:**

During intervention adoption, all stakeholders emphasized the importance of parental support, and accompanying workshops and promotional materials. Additionally, parents and teachers indicated that a shared responsibility for children’s health and nuanced framing of health messages were important. During implementation, all stakeholders needed clear guidelines and support structures. Teachers and children highlighted the importance of peer influence, social norms, and uniform application of guidelines. School staff also found further tailoring of the intervention and dealing with financial constraints important. For long-term intervention sustainment, incorporating the intervention policies into the school statutes was crucial according to health promotion professionals.

**Conclusions:**

This qualitative evaluation provides valuable insights into factors influencing the adoption, implementation, and sustainment processes of dietary interventions, such as the importance of transparent and consistent intervention guidelines, clear communication regarding the rationale behind intervention guidelines, and, stakeholders’ involvement in decision-making.

**Supplementary Information:**

The online version contains supplementary material available at 10.1186/s12889-024-18814-1.

## Background

Globally, almost one in every five children and adolescents aged 5–19 years has overweight or obesity [1]. This prevalence is specifically on the rise among people with a lower socio-economic position (SEP) [1, 2, 3]. Childhood obesity often persists into adulthood [4] and has various short- and long-term detrimental effects on children’s health [1, 5]. Many childhood obesity prevention efforts aim to stimulate healthy dietary and physical activity (PA) habits through supportive environments, health promotion policies and other intervention initiatives [6, 7]. Schools have become a popular setting for health promotion interventions as schools enable reaching many children from different SEP and cultural backgrounds, in an environment with a strong social, and potentially supportive network of teachers and peers. Every school day children spend a considerable proportion of their time at school and consume their lunch, mid-morning snacks, occasionally treats, and several drinks. Consequently, school-based interventions hold much potential to improve child health behaviors, especially regarding healthy dietary and PA habits [8, 9, 10, 11].

However, to date, the effects of different types of school-based health promotion interventions have been mixed [9, 10, 11, 12, 13, 14] and realizing effective changes among children with a lower socio-economic position seems especially challenging [15]. Moreover, research also shows that even with initial success, sustaining children’s behavioral change over time often remains challenging [16]. Often, facilitators and barriers to interventions’ adoption, implementation and sustainment processes are presented as stand-alone factors. However, in the complex reality, these factors may interact. Therefore, it should be considered to change or tackle these factors in order to more successfully adopt, implement and/or sustain school-based interventions [17]. As such, more insights are needed from all different types of actors involved in the implementation process in order to facilitate the improvement of these processes in the context of their real-world, dynamic practices and to have a more sustainable impact on daily public health practice [18, 19].

An example of a Health Promoting School intervention [10] that has been successfully embedded in practice is the Jump-in intervention [20, 21, 22]. Jump-in primarily focusses on stimulating healthy dietary and PA habits among primary school children in Amsterdam, the Netherlands [20, 23]. This intervention was first designed and implemented in 2002 and was primarily implemented at schools located in the low-SEP regions of Amsterdam, where prevalence rates of childhood overweight and obesity are often relatively high [20]. The current study aims to evaluate the adoption, implementation and sustainment processes of the dietary component of the Jump-in Health Promoting School intervention, in order to identify factors to optimize these processes within this intervention and similar Health Promoting School interventions.

## Methods

### Intervention description

Part of the larger obesity prevention program, the Amsterdam Healthy Weight Approach (AHWA [20]), Jump-in was originally designed and shown to be effective in stimulating PA [21, 22, 24]. Following this original positive evaluation, Jump-in was expanded to also include a healthy dietary component and to be further shaped after the Health-Promoting School model (HPS) [23, 25, 26]. Alongside HPS components such as “structurally involving parents” Jump-in comprises three main behavioral change intervention components: stimulating healthy dietary habits, stimulating PA and stimulating active recess play. The City of Amsterdam primarily implements Jump-in in the low-SEP areas of Amsterdam, where overweight/obesity rates are higher than the Dutch national averages. The Jump-in dietary intervention component consists of a set of healthy school nutrition policies, see Table 1. More details on Jump-in [21, 22, 27] and the AHWA [20, 28] have been published elsewhere.

**Table 1:** Description of the dietary component of the Jump-in program

**1. School nutrition policy**
Children are to only bring to and consume at school (1) a healthy mid-morning snack, consisting of fruits and/or vegetables, (2) a healthy lunch, including whole-wheat products, (3) water, tea without sugar, or milk, and (4) treats for birthdays or other festivities that are healthy, small or non-food
**2. Active parent involvement**
To encourage intervention support and compliance among parents, information meetings are organized, newsletters are distributed, and workshops on relevant themes (e.g., water, fruits and vegetables, breakfast and lunch, and treats) are conducted, such as an interactive theater show
**3. Workshops and promotional materials**
To stimulate intervention adoption, schools are encouraged to make use of educational workshops and promotional materials for children [29]
**4. Tailored support**
To guide the implementation process, each school receives support from a specialized health promotion professional (HPP) who works for the Public Health Service of Amsterdam. Additionally, a school also appoints its own internal “Jump-in coordinator”. This coordinator, the external HPP, the school’s principal and designated teachers form a joint Jump-in team. Although the main intervention goals (see (1) School Nutritional Policy) are preset, this Jump-in team tailors the intervention’s adoption, implementation and sustainment processes to the needs and culture of the school, its staff, children and parents. Typically, tailoring mainly occurs during the adoption phase, such as permitting additional whole wheat products alongside whole wheat bread, restricting beverage options to water instead of tea without sugar and milk to simplify compliance monitoring, and specifying the sequence in which policy components are initiated. During this adoption phase, schools often engage in an ‘adjustment phase’, characterized by the active utilization of promotional materials, teacher presentations, and child and parent workshops, prior to the actual implementation. HPPs actively support implementation for approximately three years. In general, this is the time frame for full intervention implementation and embedment within a school. Bi-annually, all HPPs uniformly log undertaken activities and implementation progress with this team and further fine-tune the implementation process. Eventually, all healthy policies are to be integrated into the school’s statutes and structurally implemented without external support from the City of Amsterdam or its Public Health Service

### Study design

This study used a combination of interviews and focus groups to distill lessons learned from the intervention’s design, adoption, implementation, and sustainment (or embedding). This study is part of a larger mixed methods evaluation study of the Jump-in program [27].

### Interviews and focus group discussions

Semi-structured (group) interviews and focus group discussions were conducted approximately 12 or 24 months after the start of the intervention’s implementation. Their focus was to gain insight into participants’ overall experience with the intervention and the contextual factors that (potentially) hampered or facilitated its adoption, implementation and sustainment processes. By involving such a broad range of relevant actors, we aimed to obtain different perspectives on the adoption, implementation and sustainment processes of the intervention. All interviews and focus group discussions were conducted in person within the confines of participants’ school, by two researchers in different combinations (FT and V.B., V.D., V.T. or S.J.). The interview and focus group guides were based on the implementation model of Fleuren et al.: Determinants of Innovation Model [30] with additions from previous interviews conducted by De Meij et al. [22] and Van Nassau et al. [31]. Per school, individual interviews were held with the school principal, Jump-in coordinator, two teachers, and the involved HPP. In some cases, interviews with different respondents were combined into one interview upon participants' request. We collected their views on contextual factors (e.g., socio-political, organizational, user and innovation context, and innovation strategies) across adoption, implementation, and sustainment phases. An example question was: “What factors are hindering the implementation of the Jump-in nutrition policy at this school?".

In addition, for each school, one focus group discussion was held with approximately six to eight parents of 4-to-12-year-old children and another with the same number of 8-to-12-year-old children. We decided for sessions of 6–8 individuals to both provide a variety of perspectives and allow everyone a chance to speak and share their views. However, in some cases, we were unable to reach the minimum of six participants, and we conducted a group interview instead. Discussions with parents mainly focused on the alignment of the intervention with existing school culture (socio-political context), clarity of intervention guidelines (innovation context), and their perception of the workshops and promotional materials (innovation strategies). We gathered opinions on contextual factors (e.g., socio-political, innovation and innovation strategies) across intervention phases using statements, sticky notes and plenary discussions. An example statement was:”I agree with the school restricting regulations with regards to healthy eating and drinking in the classroom, And why (not)?”. When transitioning from a focus group to group interviews, the topics remained similar, the guide was simplified, and statements and sticky notes were not used. The discussions with children mainly focused on their opinions on the intervention (innovation context) and its impact on their (school) lives (socio-political context), and their opinions on workshops and promotional materials (innovation strategies). For children, we used more creative methods such as drawings and sticky notes, along with plenary discussions. An example exercise was: "Draw your life after the Jump-in nutrition policy" after which the drawings were discussed. Detailed information on the interview guide, codebook and researcher characteristics are described in the Consolidated criteria for reporting qualitative studies questionnaire (COREQ) [32] in additional file 1 and the study protocol [27]. The topic list for the interviews and/or focus groups (in Dutch) are available on request.

### Recruitment & data collection

Schools’ enrollment in the Jump-in intervention occurred during the school year 2016–2017. Schools were eligible to participate in the evaluation study based on identical criteria to those for participation in the Jump-in intervention: schools where obesity rates exceed national averages and that are not participating in other health promotion interventions. At the time of this study, Jump-in was already (being) implemented at more than 100 other primary schools in Amsterdam. From all 10 participating schools, we recruited a convenience sample of teachers, parents and children. The schools’ usual communication channels (e.g., website, newsletters) were used to recruit children and parents, and further adjusted in consultation with the Jump-in coordinator to promote participation. Furthermore, we aimed for a diverse sample of participants, including teachers (across gender and teaching grade), parents (across gender and their child’s age), and children (across gender and age). On a few occasions, we exceeded the required subscription (i.e. two teachers, eight parents and children), enabling us to select a sample with more diversity in terms of gender, grade levels and children’s age, If the number of subscriptions allowed it, we also attempted to prevent the participation of both parent and child from the same family. The interviews and focus groups took place between November 2017 and July 2019.

### Data analysis

The interviews and focus group discussions were recorded, transcribed verbatim and analyzed in MaxQDA 2018. The latter was done independently by two researchers. We used Directed Content Analysis [33], a methodology that enabled us to build upon existing research and theories as well as distill novel codes derived from our findings. Prior to data collection, we developed a backbone coding scheme using Fleuren’s Determinants of Innovation Model [30], yet allowing for a significant degree of open coding. Per phase, this model describes determinants and processes at the level of the sociopolitical context, organization, user, and intervention/innovation, i.e., adoption, implementation, and sustainment. The comprehensive coding of all data according to this model, identifying barriers and facilitators within each phase, generated an extensive results section characterized by substantial overlap due to the abundance of data. Subsequently our approach involved thematically summarizing and analyzing the data to distill overarching themes.

## Results

In total, 13 focus groups and 39 (group) interviews were conducted, involving a total of 131 participants. Six focus groups and three group interviews were conducted with parents (Mean 74 min) and seven focus groups were held with children (M 40 min). These sessions lasted between 26 and 89 min. Additionally, individual interviews were held with seven school principals (M 44 min), seven Jump-in coordinators (M 43 min), 20 school teachers (M 28 min) and five HPPs. These interviews ranged in duration from 15 and 93 min (M 74 min). Background characteristics are shown in Table 2. Specific recurring key themes are discussed below, structured according to their relevance to the adoption, implementation and sustainment phases of the intervention. Some themes were relevant for multiple phases of the intervention. However, to avoid repetition, the themes are discussed in the phase they are most relevant to.

**Table 2:** Characteristics of teachers, parents and children

Teachers (*n* = 20)	Female (%)	90
	Teaching grade	
	1–2	8
	3–5	5
	6–8	5
	Unknown	2
Parents (*n* = 50)	Female (%)	90
	Grade child^a^	
	1–2	19
	3–5	19
	6–8	22
Children (*n* = 42)	Girl (%)	57
	Grade	
	5	5
	6	14
	7	11
	8	10
	Unknown	2

^a^Multiple parents have children enrolled across various classes, resulting in a discrepancy between the count of child groups and participating parents

### Adoption phase

#### Support and shared responsibility

The intervention dietary components were generally well-accepted and easily implemented. At times, especially the treats policy led to some resistance, because parents or teachers believed that healthy products were less festive than unhealthy treats.

Generally, parents and school staff felt a sense of shared responsibility towards children’s health. Both actors felt that schools should play a supporting role, while respecting parents’ autonomy. If they felt this was the case, parents felt more inclined to be positively engaged.

Apart from their personal beliefs, teachers’ acceptance and support for the intervention was also influenced by how they were included by the school board in the decision-making process to participate. Active participation in the decision-making process was said to increase their ownership, motivation, and encourage better implementation overall. Involving teachers and parents in deciding which intervention elements would (not) fit the school, gave them the opportunity to anticipate and respond to issues more effectively. However, some school principals and teachers expressed other viewpoints, including that a larger school size, time constraints and clear communication regarding the reasons behind policy/intervention choices decreased this willingness or desire to be part of the decision-making process.

#### Framing of the intervention

From the initial communication onward, the intervention goals, materials, and all communications explicitly aimed to convey a focus on a healthy and balanced lifestyle. However, some parents and teachers mainly perceived it as an obesity-focused program, which led to fears of it being stigma-inducing. This was mentioned as a barrier for parental support and consequently for successful implementation, because many parents and teachers considered it important to teach children that enjoying an occasional unhealthy treat is acceptable as long as it is integrated into an overall healthy diet.

*“I believe you should teach children the nuance, [...] now it is ‘chocolate bad’. No, chocolate is not always bad. That is what I want to teach children […] sometimes you may cheat”* - Teacher

#### Workshops, activities and materials

To facilitate a more effective implementation of the nutritional policy, the intervention provided several educational workshops, and promotional and educational materials to children and parents throughout the adoption phase. The intervention´s activities and materials, especially the interactive parent theatre, were generally well-received and facilitated active engagement and support among all actors.

Teachers also valued the interactive format and educational quality of the workshops. They felt the workshops were educational and created support, which facilitated adoption and implementation processes. The same was said about the promotional materials. Children found them fun and engaging, while parents believed they contributed to creating a positive social norm towards healthy dietary habits.

However, some promotional materials were viewed as redundant and recurring criticism was expressed regarding most of the materials being cheap plastics and hence environmentally unfriendly. For example, the intervention’s “birthday treat guidelines” often led to children treating their classmates to small toys instead of food. Parents and teachers felt it would be good if such guidelines were to represent a more holistic view that integrates both health and environmental sustainability. Another point for improvement was the further tailoring of materials to children’s cognitive abilities to fit a broader age range of children.

In addition, parents and teachers pointed out that workshops were generally attended by those parents that were already aware of the importance of stimulating a healthy lifestyle among their children. Therefore, they indicated that involvement of a more diverse range of families should be a priority, e.g., via digital mobile school applications.

### Implementation phase

#### Peer influence and social norms

Children, parents and teachers noted that peer influence in class (mostly positively) impacted intervention adherence. This became apparent during, for instance, the use of promotional materials that visually expressed a social norm. “So-called” water trackers, which showed the percentage of children adhering to the water drinking policy, and were consequently perceived to stimulate water consumption. Especially among younger children, teachers said it stimulated them to adhere to the healthy nutrition policies. Some parents noticed how their child(ren) only wanted to bring water to school, because otherwise they would*’ruin the water tracker for the rest of the class’*. However, in some cases, these norm setting techniques were said to be counterproductive in older children.

*“I notice there is a lot of social control. The children check it themselves or they come to me ‘huh I brought this [non-compliant food]’. I’m actually surprised how strict they are.”* Teacher

#### Teacher beliefs and practices

Teachers indicated their main priority was having a good relationship with parents and providing children with the best education possible, not being a health promoter enforcing nutritional guidelines. Forcing them to ‘be police officers’ endangers that relationship, which teachers found unacceptable. Some teachers therefore occasionally allowed children to bring foods they were not supposed to, because they felt unable to combine to roles of a good educator and health promotor. Also, some teachers felt that the intervention content was not congruent with their personal view on health. For instance, some believed a child’s birthday should be a celebration, and did not feel it was right to deny them an unhealthy treat. This sometimes led to intervention dilution.

*“I feel like not all colleagues support it [the intervention] to the same extent. Especially for lunch, people say ‘but I’m not gonna check that, this is not my responsibility’.”* Teacher

#### Perceived fairness

According to several interviewed teachers and children, acceptance of and commitment to the intervention depended on the transparent, consistent enforcement of the guidelines. When “the rules” applied to everyone, including their teachers, children accepted them and agreed with them. If not, they perceived this as “really unfair”, which made them resistant to accept the guidelines.

“*The teachers tell us to bring healthy birthday treats, but then they receive chocolates as treats. They just tell us ‘if you want sweet treats you should become a teacher yourself’; it is really unfair when we can’t have any sweets and they can*” - Child

#### Healthy eating on a budget

All interviewed actors except for children indicated that some parents had mixed feelings about the intervention due to budget constraints. Sometimes the healthy policies created difficult situations for teachers. They did not feel comfortable obliging children to bring fruits, vegetables, and whole-wheat products, which are perceived as expensive, knowing that many families have to deal with financial difficulties.

“*Parents feel ashamed for their poverty and we as teachers don’t want to create uncomfortable situations by obliging these parents to bring expensive fruits to school*.” – Teacher

Conversely, the water-only policy was often popular, as was the provision of materials such as lunchboxes, water bottles and fruit holders. Not only did it save money, but some parents mentioned children could no longer derive social status from brand materials.

#### Tailoring the intervention

Schools (i.e., school principals, Jump-in coordinators and teachers) considered it essential to be able to tailor the intervention to schools’ needs and culture. Some appreciated how they could implement the policies in several phases over longer periods of time, while others wanted to implement everything at once. However, some HPPs, school principals, Jump-in coordinators and teachers stated the importance of finding a balance between ensuring the nutrition policies fit the wishes of parents, children and teachers, and providing clarity to maintain fidelity. Some parents expressed a preference for guidelines that allowed for more variety, creativity and cultural adaptations. However, allowing for such adaptations was said to make it increasingly hard for teachers to monitor adherence to the guidelines. Schools therefore often allowed for a relatively limited number of options for foods that could be brought school, because they valued clarity over variety and diversity. This was experienced as a difficult balance at times, because parents and children sometimes expressed finding the policies overly restrictive and boring. Several parents stated that recurring discussions on whether certain consumptions were healthy enough to meet the guidelines could have been prevented with more transparency on why the nutrition policies were designed that way.

*“ Brown bread is very limiting for a school with so many different nationalities. (…) I want my kids to eat soup, wholegrain rice dishes and tortilla wraps because there is so much more than bread.” - Parent*

#### Additional support for implementation and sustainment

Despite generally being satisfied with the intervention, some teachers stated that the received implementation support could be improved.

*Teachers* experienced a shortage of time due to “additional projects” surrounding health promotion. It would benefit them to have better work protocols with clear work instructions and support regarding e.g., how to deal with parents that disagree with the healthy school policies, how to best deal with related conflicts or resistance, how to monitor adherence to the new policies and how to best deal with children bringing consumptions that violate the nutrition policies. Furthermore, teachers indicated that children value and are very aware of whether their teachers ‘practice what they preach’, so several teachers stated they welcomed practical support on how to set the right example as a role model.

*School principals* indicated needing better support in keeping their teaching staff and parents engaged. This meant providing sufficient opportunities for teachers to discuss concerns, give and receive inter-collegial support and to keep reminding them of the school policies. However, with teachers’ busy schedules in mind, and the fact that teachers are first and foremost *educators and not professional health promoters*, school principals indicated they also needed help via, e.g., work protocols or practical guidelines on how to best support their teaching staff in combining their roles as educators and health promoters.

*Parents* often said fresh fruits and vegetables are more expensive than unhealthy alternatives and that birthday treats and celebrations are often associated with unhealthy foods. Support on how to mix ‘healthy’ and ‘festive’ was said to be needed. Only few parents were aware of the already existing intervention tool ‘birthday treat book’. In addition, parents indicated they would be better supported with an easily accessible, central place to express concerns and ask questions.

Lastly, several *HPPs* indicated they struggled with their two-fold agenda, i.e., getting schools enthusiastic about adopting the new healthy nutrition policies, while also empowering them to feel ownership and take responsibility for those changes. They were often the driving force behind important changes. However, this role as change leaders simultaneously caused schools not to consider these changes as ‘theirs’, but rather as ‘Jump-in rules’. Trying to balance taking on this supportive role with simultaneously encouraging a school to take charge and feel ownership was experienced as particularly challenging by the HPPs. The importance of schools feeling ownership was emphasized by the process of structurally embedding the intervention in a school. This is done by integrating all nutrition policies into the school’s curriculum and statutes, which is intended to ensure long-term sustainment of the nutrition policies. Enforcement of these policies often watered down when a school came under new management. Therefore, HPPs stressed that developments regarding intervention sustainment are still needed.

### Sustainment of the intervention

#### Innovation

Although data collection took place before the sustainment phase and hence insights regarding sustainment were limited, we deemed these insights important to include. The intervention aims to facilitate sustainment by integrating its content (i.e., the nutrition policies) into the school statutes. HPPs indicated that this worked well most of the times, but sometimes did not prove sustainable in the long term. It was recurrently indicated that innovations were needed to create better intervention sustainment. Some interviewees stated that intervention acceptance, ownership and habit formation were important conditions for a sustainable implementation. Additionally, the HPPs said they needed more tools to adapt the intervention in real life together with the main actors without compromising the intervention’s effective elements.

## Discussion

This study presents a qualitative evaluation of the adoption, implementation and sustainment processes of the Jump-in dietary intervention. We collected perspectives from children, parents, teachers, school principals, at-school Jump-in coordinators and municipal HPPs in a total of 13 focus groups and 39 (group) interviews, including a total of 131 participants. Overall, the intervention was well-accepted and received with enthusiasm among all stakeholders, yet various opportunities for improvement came to light.

Regarding the *adoption phase,* participants felt it was important for the HPPs and school principals to be transparent about the intervention’s goals and processes. It also stood out that parents, children, and teachers wanted to have a voice in the decision-making processes regarding intervention content and implementation. Although each school’s Jump-in team included several stakeholders (generally a HPP, school principal, a local Jump-in coordinator and several teachers), parents and teachers indicated they wanted to be better included in the decision-making process. This corresponds with previous recommendations to design and implement health promotion interventions together with all end-users for more appropriate, better fitting and more effective interventions [34].

The main lessons regarding the *implementation phase* were that having clear, transparent nutrition rules that apply to all children as well as their teachers (i.e., role modelling) and having a clear way to unanimously communicate and enforce those rules, were considered essential for their sustained support. Allowing children to bring a limited selection of “healthy” food options to school was experienced as overly restrictive and boring, especially in a multicultural city such as Amsterdam. However, having to discuss these rules with children and parents, teachers felt the pressure to act as “the health police”, which, in their view, endangered their relationship with parents as well as the intervention’s sustainability. The role of health promotor sometimes conflicted with their role as educator. Other studies have also reported difficulties when merging the roles of health promotor and educator in school health promotion [35], stating the need to align health promotion efforts with educational goals [36] and the need for innovations and support structures for sustainable success in comprehensive HPS efforts in real-world contexts [37]. It therefore seems vital to make health promotion a core business for schools, align efforts with national policies, use local data to show their need and effectiveness, and provide high-quality, pragmatic and accessible staff training [38].

For the *sustainment phase*, integration into the school statutes was often noted as being crucial to the sustainable implementation of the Jump-in intervention. Efforts to stimulate sustainable implementation start at intervention design and adoption, e.g., providing support that fits involved participants/actors within the structures, workings, and culture of their school’s system.

### Interacting barriers and facilitators

Many of the factors (either barriers or facilitators) that we found in our study did not act as stand-alone, individual influences, but rather as parts of specific, sometimes elaborate, interactions impacting the program’s adoption, implementation or sustainment. For example, some parents wished for an expansion of the healthy policies by including a selection of foods and drinks that were considered more culturally inclusive, which simultaneously complicated the teacher’s role that included checking whether all children adhered to the school policies. Also, teachers emphasized the importance of educating children about nuances, such as instances where exceptions to the guidelines are permissible. However, children perceived differences in how teachers enforced the nutrition guidelines as unfair. Qualitative evaluations of health promotion interventions often present lists of barriers and facilitators [18, 30, 39], yet rarely note their complex interactions, which sometimes leads to oversimplified recommendations. Instead, Darlington and colleagues describe five types of interactions: hindering, moderating, counterbalancing, enabling and neutral [35]. Such distinctions may help report and understand how factors are positioned within more complex mechanisms of change. With our thematic analyses, we aimed to understand the influence of certain barriers and facilitators, in a broader context. Such considerations and views could benefit the development of context- and phase-specific strategies to help implement interventions in a real-world setting [18].

### Tailoring while maintaining fidelity and effectiveness

A recurring theme in our study was the challenge to implement the intervention as intended (i.e., maintaining intervention fidelity) while simultaneously allowing for, and even stimulating, local adaptation, ownership, and shared responsibility. Allowing for local adaptations makes an intervention more (culturally) appropriate, stimulating feelings of ownership and intrinsic motivation among actors, which may also benefit effectiveness and long-term sustainment. For HPPs this was challenging, confirming HPS intervention studies [9, 40, 41]. Jump-in’s HPPs aimed to adapt the intervention to the local context together with relevant stakeholders while building on evidence-based behavioral change techniques [42, 43] and their peer-to-peer learning network. In addition, there is a need for tools that HPPs can use to distinguish crucial versus adaptable intervention components, so that the intervention’s effectiveness is secured, e.g., Intervention Mapping Adapt [44, 45]. Taking the lead in the implementation processes, however, also hampered the process of ensuring schools feel the sense of ownership and responsibility required to create long-term embedment of the desired system changes [17, 41]. We recommend more research on practical tools for HPPs that enable school actors to feel ownership while simultaneously facilitating smooth implementation processes.

### Schools as complex systems

Several hampering factors might be better understood when viewed in the context of a systems approach. For instance, challenges encountered by some teachers in playing the role of “police officers”. Each school represents a unique context, and it is therefore also important to address contextual differences between schools. For example, some teaching teams perceived it as a barrier when they were not involved in the decision-making process, whereas teachers at other schools preferred a small group to handle this, as long as they were kept updated. Additionally, variations were observed in the extent to which parents accepted information from health professionals as accurate. It is therefore crucial to consider the context within schools. Accomplishing sustained changes in schools – as in most complex adaptive systems – often requires coordinated, complementary actions on different socioecological levels [17, 25, 26, 28]. Viewing schools as such in public health promotion efforts can advance the evolution of the symbiosis between health and education [9, 17, 46]. Looking at the implementation processes via the systems view of the Action Scales Model (ASM) [47] shows how aiming to change certain system structures without also dealing with root system goals is unlikely to lead to durable, effective changes. This model states that a system is shaped by a combination of concrete, visible elements (i.e., actions and structures, the tip of the iceberg) and the underlying beliefs and goals that shape them. Schools, for example, revolve around the belief that providing a child with the best possible education is vital to its success in life. Given that belief, and consequent goals, structures, and actions (or: events) naturally emerge to achieve those goals. Therefore, changing certain system structures (e.g., implementing certain healthy nutrition policies in schools) is more likely to succeed when aligned with the underlying system beliefs and goals (e.g., education). Requiring teachers to place their role of health promotor above that of educator in a system that revolves around providing education is unlikely to be successful and sustainable. However, results indicate this is what happens when teachers are expected to argue with parents over healthy nutrition guidelines, thereby endangering their parent-teacher relationship. Creating more lasting impact is likelier to succeed with a simultaneous integration of intervention change objectives focused on targeting underlying systems goals and relevant actors’ beliefs. It would help, for instance, to get school boards to prioritize children’s health alongside their educational achievements. This way, taking such a *systems perspective,* can provide new insights into potential new leverage points to intervene on and help to create more durable, impactful health promotion initiatives. Therefore, future research could benefit from integrating such approaches during the initial phases of HPS evaluations.

### Strengths and limitations

A strength of the current study is its large size. With 39 individual and group interviews and 13 focus groups with a total of 131 participants, it provides a broad range of perspectives from all main actors involved in the intervention’s adoption, implementation, and sustainment processes in a real-world setting. Shaping the data collection and analysis with a clear theoretical framework also added to the study’s strength. In addition, the qualitative evaluation was generally carried out one to two years after intervention adoption, which provided sufficient time to experience recurring, structural barriers and lessons learned. Yet, this timeframe was most of the times not sufficient to capture sustainment.

The study also had certain limitations. Firstly, as is generally the case in research, parents that were willing to participate were potentially more likely to have relatively extreme opinions on the intervention leading to an overrepresentation of their strong views, a phenomenon known as self-selection bias [48]. In addition, despite efforts to mainly select parents from a low SEP, informal data on occupation suggest parents from high SEP backgrounds also participated in the focus groups discussions. However, due to the sampling size this influence is likely minimal and still allowed for obtaining a representative view on the perspectives from both low and high socioeconomic position (SEP) groups.

## Conclusions

This qualitative evaluation provides novel insights and lessons about the adoption, implementation, and sustainment processes of the Jump-in dietary intervention. Including all stakeholders in the decision-making and implementation processes appeared to be key to the intervention’s early adoption and acceptance. Transparency and uniformity of intervention guidelines appeared important for parental and child support. Parents requested better communication about the reasons behind guidelines. Furthermore, identified key challenges included understanding how barriers and facilitators operate and interact within more intricate mechanisms of change, maintaining a balance between intervention fidelity and tailoring the intervention to the local context, and successfully implementing and structurally embedding a health promotion initiative within a larger system (i.e. a school) that prioritizes promoting good education over health as its primary goal [49, 50, 51].

### Supplementary Information

Supplementary Material 1.

Supplementary Material 2.

## Data Availability

Data can be obtained from the corresponding authors upon reasonable request.

## Abbreviations

HPP: Health promotion professional
HPS: Health promoting school
AHWA: Amsterdam healthy weight approach
PA: Physical activity
SEP: Socio-economic position
DoI: Determinants of innovation model
