# Supplementary material for: Qualitative multi-stakeholder evaluation of the adoption, implementation and sustainment of the school-based dietary intervention “Jump-in”
Source: BMC Public Health. 2024 May 17;24:1337. doi: 10.1186/s12889-024-18814-1 (PMC11102190; doi:10.1186/s12889-024-18814-1)
Supplement: Supplementary file 1 — Supplementary Material 1. [file 12889_2024_18814_MOESM1_ESM.pdf]

Additional file 1

Appendix I COREQ checklist
Consolidated criteria for reporting qualitative studies (COREQ): 32-item checklist

NO	Item	Description
**Domain 1: Research team and reflexivity**		
Personal characteristics		
1.	Interviewer/facilitator	The executive researcher (F.T.) conducted most of the interviews and all focus groups. Interns conducted some of the interviews (V.D., V.T. and S.J.) and data analysis (II) with the help of an executive researcher (F.T. or V.B.)
2.	Credentials	F.T. = MSc<br>V.D. = BSc<br>V.T. = BSc<br>S.J. = BSc<br>I.I.= MSc<br>V.B. = PhD
3.	Occupation	F.T. = PhD student<br>V.D. = MSc student<br>V.T. = MSc student<br>S.J. = MSc student<br>I.I.= MSc student<br>V.B. = senior researcher
4.	Gender	F.T. = female<br>V.D. = female<br>V.T. = female<br>S.J. = female<br>I.I. = female<br>V.B. = male
5.	Experience and training	F.T. = MSc degree and professional experience<br>V.D. = MSc training<br>V.T. = MSc training<br>S.J. = MSc training

		I.I. = MSc training<br>V.B. = PhD degree and professional experience
Relationship with participants		
6.	Relationship established	Since this is an evaluation study conducted after the initial effectiveness study, we had already established relationships with some actors (HPPs, school principals) but not with others (parents, teachers, children) prior to the study commencement.
7.	Participant knowledge of the interviewer	At the start of the interview the researcher introduced herself with her name, background, occupation and the reason for doing the research.
8.	Interviewer characteristics	The executive researcher had no competing interest in the research. The researchers did not state their personal opinions or believes during the interviews and focus groups.
**Domain 2: study design**		
Theoretical framework		
9.	Methodological orientation and Theory	The study was based on directed content analysis, as described by Hsieh & Shannon.
Participant selection		
10.	Sampling	HPPs = purposive selection;<br>Jump-in coordinator = purposive selection;<br>School principal = purposive selection;<br>Teachers = convenience selection;<br>Parents = convenience selection;<br>Children = convenience selection
11.	Method of approach	HPPs: face-to-face, email;<br>Jump-in coordinator: telephone, email;<br>School principal: email, via Jump-in coordinator;<br>Teachers: face-to-face, email, via Jump-in coordinator;<br>Parents: letter, via Jump-in coordinator;<br>Children: face-to-face, letter, via Jump-in coordinator
12.	Sample size	Total interviewees: 131<br>HPPs: 5

		Jump-in coordinators: 7<br>School principals: 7<br>Teachers: 20<br>Parents: 50<br>Children: 42
13.	Non-participation	Due to logical constraints one HPP and two principals did not participate. For the same reason, some schools decided not to organize a focus group discussion for parents (n=1) or children (n=3).
Setting		
14.	Setting of data collection	Primary schools (Jump-in coordinator, school principal, teachers, parents, children). Public Health Service (HPP).
15.	Presence of non-participants	Besides participants and researchers nobody was present, except for occasional disruptions. For example: disruptions by a colleague who had to get something out of the classroom or by principals who checked in for a minute if everything was going well.
16.	Description of sample	The sample included schools located in disadvantaged neighborhoods of Amsterdam, with ethnic minority groups with a low SES.
Data collection		
17.	Interview guide	F.T. and V.D. together set up the interview guides. They were not pilot tested, yet adapted iteratively after each interview.
18.	Repeat interviews	No repeat interviews were carried out.
19.	Audio/visual recording	All interviews were audio recorded.
20.	Field notes	Field notes were made during and after data collection. However, the researchers used interview transcriptions for this paper's analysis.
21.	Duration	HPP interview: 00:36:13 – 01:33:19 (mean 01:13:36)<br>Jump-in coordinator interview: 00:25:38 – 01:04:45 (mean 00:42:45)<br>School principal interview: 0:36:36 – 00:57:17 (mean 00:43:02)<br>Teachers (group) interview: 00:15:06 – 00:36:38 (mean 0:27:41)

		Parent focus group discussion: 00:51:38 – 01:28:55 (mean 1:14:26<br>Child focus group discussion: 00:26:26 – 00:49:12 (mean 00:39:44)
22.	Data saturation	Data saturation was discussed and the interview guide was adapted accordingly.
23.	Transcripts returned	Transcripts were not returned to participants for comments or corrections.
**Domain 3:<br>Analysis and findings**		
Data analysis		
24.	Number of data coders	4 (F.T., S.J., V.D., I.I.)
25.	Description of the coding tree	The coding tree was based on the framework by Fleuren et al. (2004), see Appendix II.
26.	Derivation of themes	The coding tree was guided by the Determinants of innovation model by Fleuren et al. (2004) after open coding the data.<br>When text fragments could not be assigned to the predetermined coding framework during data analysis, additional codes were created. This process was performed independently by one researcher (F.T., V.D., S.J. or I.I) and checked independently by another (F.T. or V.B.).
27.	Software	MAXQDA 2018
28.	Participant checking	Participants did not provide feedback on the findings.
Reporting		
29.	Quotations presented	Participant quotations were presented to illustrate the themes and findings. Quotations were not identified with a participant number.
30.	Data and findings consistent	Yes.
31.	Clarity of major themes	Yes, see results.
32.	Clarity of minor themes	Partially. We emphasized the major themes in the results section.
