# Supplementary material for: Qualitative multi-stakeholder evaluation of the adoption, implementation and sustainment of the school-based dietary intervention “Jump-in”
Source: BMC Public Health. 2024 May 17;24:1337. doi: 10.1186/s12889-024-18814-1 (PMC11102190; doi:10.1186/s12889-024-18814-1)
Supplement: Supplementary file 2 — Supplementary Material 2. [file 12889_2024_18814_MOESM2_ESM.pdf]

Additional file 2

Appendix II Code system

Background information
1.Satisfaction
2.Degree of implementation
3. Nice quotes
4. Dissemination phase
4.1 Socio-political context
4.1a Willingness of parents/child to cooperate
4.1b Awareness of health benefits parents/child
4.1c Financial burden/benefits of the food policy parents/child
4.1d Innovation fits into existing rules, regulations
4.1e Perceived responsibility (parents)
4.2 Organization level
4.2a Decision making process
4.2b Organizational policies & priorities
4.2c Oranizational size
4.2d Collaboration with partnerships and networks
4.2 e Collaboration within organization
4.2f Staff turnover (high, average, low)
4.2g Staff capacity
4.2h Plans for continuation food policy in the future
4.3 User level (teacher)
4.3a Support/pressure form colleagues
4.3b Support/pressure form director/JI coordinator
4.3c Knowledge
4.3d Modeling
4.3e Self-efficacy
4.3f Ownership/commitment/motivation
4.3g Work related stress (enough time)
4.3h Intention to continue JI policy
4.3i Attitude (eg. ethics, contradictory, goals)

4.3j Perceived responsibility (teacher/school)
4.3k (innovation fits in the perceived task orientation)
4.4 Innovation level
4.4a Clarity procedure/ guidelines of food policy
4.4a.1 Teachers
4.4a.2 Parents
4.4a.3 Children
4.4b Compatibility with regular tasks and curriculum
4.4c Extent to which the innovation is appealing to use
4.4d Relevance of the innovation for children/parents
4.4e Inclusion-exclusion criteria
4.4f (visibility of results)
4.5 Innovation strategy
4.5a Financial resources
4.5a.1 Reimbursement for health professionals
4.5b Resourches implementing innovation
4.5b.1 Workshops parents
4.5b.2 Workshop children
4.5b.3 Workshop teachers
4.5b.4 Materials
4.5b.5 Communication resources (bv. newsletter, app)
4.5c Administrative support (JI advisor/GGD)
4.5d Tailoring Jump-in school
4.5e Opinion leader influences opinions or other organisations
5. Adoption phase
5.1 Socio-political context
5.1a Willingness of parents/child to cooperate
parent attitude
Child attitude
5.1b Awareness of health benefits parents/child
5.1c Financial burden/benefits of the food policy parents/child
5.1d Innovation fits into existing rules, regulations

- 5.1e Perceived responsibility (parents)
- 5.1f Changing society

5.2 Organization level

- 5.2a Decision making process
- 5.2b Organizational policies & priorities
- 5.2c Organizational size
- 5.2d Collaboration with partnerships and networks
- 5.2e Collaboration within organization
- 5.2f Staff turnover (high, average, low)
- 5.2g Staff capacity
- 5.2h Plans for continuation food policy in the future

5.3 User level (teacher)

- 5.3a Support/pressure form colleagues
- 5.3b Support/pressure form director/JI coordinator
- 5.3c Knowledge
- 5.3d Modeling
- 5.3e Self-efficacy
- 5.3f Ownership/commitment/motivation
- 5.3g Work related stress (enough time)
- 5.3h Intention to continue JI policy
- 5.3i Attitude (e.g. ethics, contradictory, goals)
- 5.3j Perceived responsibility (teacher/school)
- 5.3k (innovation fits in the perceived task orientation)
- 5.3l1 lijn tussen docenten

5.4 Innovation level

- 5.4a Clarity procedure/guidelines of food policy
  - 5.4a.1 Teachers
  - 5.4a.2 Parents
  - 5.4a.3 Children
- 5.4b Compatibility with regular tasks and curriculum
- 5.4c Extent to which the innovation is appealing to use
- 5.4d Relevance of the innovation for children/parents

5.4e Inclusion-exlclusion criteria
5.4f (visibility of results)
5.5 Innovation strategy
5.5a Financial resources
5.5a1 Reimbursement for health professionals
5.5b Resourches implementing innovation
5.5b.1 Workshops parents
5.5b.2 Workshops teachers
5.5b.3 Workshops children
5.5b.4 Materials
5.5b.5 Communication resources (bv. newsletter, app)
5.5c Administrative support (JI advisor/GGD)
5.5d Tailoring Jump-in school
5.5e Opinion leader influences opinions or other organisations
6. Implementation phase
6.1 Socio-political context
6.1a Willingness of parents/child to cooperate
6.1b Awareness of health benefits parents/child
6.1c Financial burden/benefits of the food policy parents/child
6.1d Innovation fits into existing rules, regulations
6.1e Perceived responsibility (parents)
6.1f Changing society
6.2 Organization level
6.2a Decision making process
6.2b Organizational policies & priorities
6.2c Organizational size
6.2d Collabaration with partnerships and networks
6.2e Collaboration with organization
6.2f Staff turnover (high, average, low)
6.2g Staff capacity
6.2h Plans for continuation food policy in the future
6.3 User level (teacher)

6.3a Support/pressure from colleagues
6.3b Support/pressure from director/JI coordinator
6.3c Knowledge
6.3d Modeling
6.3e Self-efficacy
6.3f Ownership/commitment/motivation
6.3g Work related stress (enough time)
6.3h Intention to continue JI policy
6.3i Attitude (eg. ethics, contradictory, goals)
6.3j Perceived responsibility (teacher/school)
6.3k (innovation fits in the perceived task orientation
6.3l 1 lijn tussen docenten
6.4 Innovation level
6.4a Clarity procedure/ guidelines of food policy
6.4a.3 Children
6.4a.2 Parents
6.4a.1 Teachers
6.4b Compatibility with regular tasks and curriculum
6.4c Extent to which the innovation is appealing to use
6.4d Relevance of the innovation for children/parents
6.4e Inclusion-exclusion criteria
6.4f (Visibility of results)
6.5 Innovation strategy
6.5a Financial resources
6.5a.1 Reimbursement for health professionals
6.5b Resourches implementing innovation
6.5b.1 Workshops parents
6.5b.2 Workshops children
6.5b.3 Workshops teachers
6.5b.4 Materials
6.5b.5 Communication resources (e.g. newsletter, app)
6.5c Administrative support (HPP/GGD)

6.5d Tailoring Jump-in school
6.5e Opinion leader influences opinions or other organizations
7. Continuation phase
7.1 Socio-political context
7.1a Willingness of parents/ child to cooperate
7.1b Awareness of health benefits parents/child
7.1c Financial burden/benefits of the food policy parents/child
7.1d innovation fits into existing rules, regulations
7.1e Perceived responsibility (parents)
7.1f Changing society
7.2 Organization level
7.2a Decision making process
7.2b Organizational policies & priorities
7.2c Organizational size
7.2d Collaboration with partnerships and networks
7.2e Collaboration within organization
7.2f Staff turnover (high, average, low)
7.2g Staff capacity
7.2h Plans for continuation food policy in the future
7.3 User level (teacher)
7.3a Support/pressure form colleagues
7.3b Support/pressure from director/JI coordinator
7.3c Knowledge
7.3d Modeling
7.3e Self-efficacy
7.3f Ownership/commitment/motivation
7.3g Work related stress (enough time)
7.3h Intention to continue JI policy
7.3i Attitude (e.g. ethics, contradictory, goals)
7.3j Perceived responsibility (teacher/school)
7.3k (innovation fits in the perceived task orientation)
7.3l 1 lijn tussen docenten

7.4 Innovation level
7.4a Clarity procedure/ guidelines of food policy
7.4a.1 Teachers
7.4a. 2 Parents
7.4a. 3 Children
7.4b Compatibility with regular tasks and curriculum
7.4c Extent to which the innovation is appealing to use
7.4d Relevance of the innovation for children/parents
7.4e Inlcusion-excluion criteria
7.4f (visibility of results)
7.5 Innovation strategy
7.5a Financial resources
7.5a.1 Reimbursement for health professionals
7.5b Resourches implementing innovation
7.5b.1 Workshops parents
7.5b.2 Workshops children
7.5b.3 Workshops teachers
7.5b.4 Materials
7.5b.5 Communication resources (e.g.. newsletter, app)
7.5c Administrative support (HPP/GGD)
7.5d Tailoring Jump-in school
7.5e Opinion leader influences opinions or other organizations
